# *Streptococcus pneumoniae* carriage and antibiotic susceptibility among Indonesian pilgrims during the Hajj pilgrimage in 2015

**DOI:** 10.1371/journal.pone.0246122

**Published:** 2021-01-26

**Authors:** Kuntjoro Harimurti, Siti Rizny Fitriana Saldi, Esthika Dewiasty, Thafsin Alfarizi, Melzan Dharmayuli, Miftahuddin Majid Khoeri, Wisiva Tofriska Paramaiswari, Korrie Salsabila, Wisnu Tafroji, Carolina Halim, Qin Jiang, Amgad Gamil, Dodi Safari

**Affiliations:** 1 Clinical Epidemiology and Evidence-Based Medicine (CEEBM) Unit, Cipto Mangunkusumo Hospital/Faculty of Medicine Universitas Indonesia, Jakarta, Indonesia; 2 Department of Internal Medicine, Faculty of Medicine Universitas Indonesia/Cipto Mangunkusumo Hospital, Jakarta, Indonesia; 3 Center of Hajj Health, Ministry of Health, Jakarta, Indonesia; 4 Eijkman Institute for Molecular Biology, Jakarta, Indonesia; 5 Pfizer Indonesia, Jakarta, Indonesia; 6 Pfizer Inc., Collegeville, Pennsylvania, United States of America; 7 Pfizer Inc., Emerging Markets Medical & Scientific Affairs, Dubai, UAE; Nitte University, INDIA

## Abstract

The Hajj is an annual pilgrimage to Mecca and one of the largest gathering of people in the world. Most Indonesian pilgrims are senior adults and elderly adults, who are more prone to acquire infections during the Hajj ritual. The aims of this study are to investigate the dynamics of *Streptococcus pneumoniae* colonization and to investigate antibiotic susceptibility of pneumococcal strains in Indonesian pilgrims. This was a prospective multi-site longitudinal study in Indonesian hajj pilgrims aged >18 years old in the year 2015. Nasopharyngeal swabs were collected from the same subject before departure and upon arrival at the airport. *S*. *pneumoniae* was identified using conventional and molecular approach, while antibiotic susceptibility was determined using a disk diffusion method. Among 813 Hajj pilgrims who were enrolled from five sites in this study, the prevalence of *S*. *pneumoniae* carriage rates before- and after-the Hajj were 8.6% (95% CI 6.7–10.5%) and 8.2% (95% CI 6.4–10.1%), (*p value*: 0.844) respectively. Serotype 16F, 6A/6B, 3, 18, and 23F were the five most prevalent serotypes before Hajj, whereas serotypes 3, 34, 13, 4, and 23F were the most prevalent serotypes after Hajj. Serotype 3 was identified as most acquired serotype during Hajj in Indonesian pilgrim. There was an increase in the percentage of isolates susceptible to co-trimoxazole after Hajj (42.9% versus 57.4%). The study provided an overview of the change of dynamics of *S*. *pneumoniae* serotype acquisition in Indonesian Hajj Pilgrims. Along with data of vaccination serotypes coverage and antimicrobial susceptibility, these findings may contribute to recommendation of vaccination and treatment policies in the future.

## Introduction

The Hajj, an annual pilgrimage to Saudi Arabia, is one of the largest annual gatherings of people in the world. During Hajj, there are more than two million people gathered in the pilgrimage journey [1]. There were 1,952,817 pilgrims during the 2015 Hajj, from which 1,384,941 foreign pilgrims originated from 164 countries [2]. Indonesia sent 168,800 pilgrims to Saudi Arabia in 2015 [3]. Around half of medical conditions requiring visit to a health facility during Hajj were due to respiratory syndromes [4]. As the cause of death of Indonesian pilgrims during Hajj, approximately 28% was attributed to acute respiratory infections [5]. Moreover, according to the study conducted in three tertiary hospitals in Mecca during the Hajj pilgrimage in 2005, the most frequent cases of pneumonia were reported from Indonesia (18.4%), followed by Saudi Arabia (17.1%) and Pakistan (11.8%) [6]. A study among Hajj pilgrims in 2016 reported that 18.0% (95% CI 13.9–23.1%) of community-acquired pneumonia cases were positive for *S*. *pneumoniae* [7].

Among the pilgrims, a large number have been identified with co-morbidities and risk factors of acquisition of pathogen microorganisms [8]. Benkouiten et al. reported that there was an increase in acquisition of *S*. *pneumoniae* carriage in a cohort of pilgrims returning from the 2012 Hajj pilgrimage (Pre-Hajj = 7.3% versus Post-Hajj = 19.5%; p = 0.001) [8]. *S*. *pneumoniae* often colonize the mucosa and could cause airborne transmission through coughing or sneezing, thus increasing the risk of transmission in crowded places where people gather for a long period such as Hajj ritual. Cross-sectional and longitudinal studies from Saudi Arabia and France done before and after Hajj showed a significant increase of pneumococcal carriage after the pilgrimage [8, 9].

Currently, the pneumococcal vaccination, either pneumococcal conjugate vaccine (PCV) or pneumococcal polysaccharide vaccine (PPV) is not part of mandatory vaccination for pilgrims. Moreover, the pneumococcal vaccination serotype coverage remains unknown due to the limited data on the distribution of *S*. *pneumoniae* serotypes in Indonesia. Therefore, this study was conducted to describe the dynamic of *S*. *pneumoniae* acquisition, profile of its serotypes, as well as antibiotic susceptibility of pneumococcal strains in Indonesian hajj pilgrims 2015.

## Materials and methods

### Study population

A prospective multisite longitudinal study to obtain the prevalence and serotype distribution of *S*. *pneumoniae* in Indonesian hajj pilgrims. Specimen collection were performed before the departure from Indonesia to Saudi Arabia (Pre-Hajj) and immediately upon arrival in Indonesia (Post-Hajj) from the same subjects.

This study was approved by The Research Ethics Committee of Faculty of Medicine, Universitas Indonesia/Dr. Cipto Mangunkusumo General Hospital, Jakarta, Indonesia (No. 608/UN2.F1/ETIK/2015). Each participant signed the written informed consent prior to enrolment.

The Indonesian Hajj pilgrims assembled in their pre-selected departure points 24 hours prior to departure where they completed the final medical check-up and provided pocketbook describing pilgrim’s health conditions, medications and vaccination status [5]. Each day, the departure points managed and facilitated departure group(s) consisted of 300–450 pilgrims accompanied by the medical team. Upon their return in Indonesia, each group returned to their corresponding departure points for health screening. Pre-Hajj data were collected at the pre-departure final medical check-up period and post-Hajj data were collected at the post-arrival health screening.

**Fig 1:**
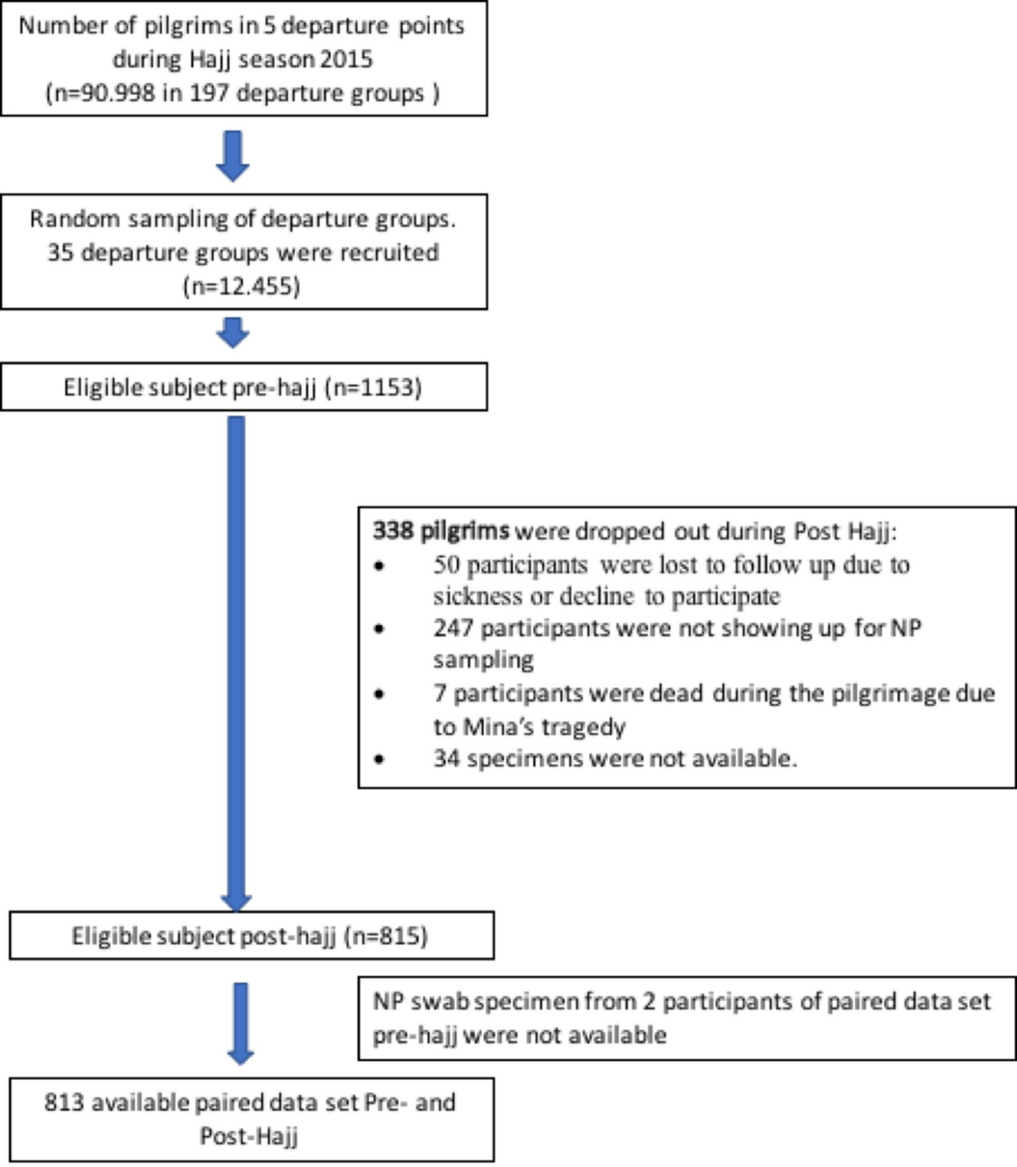
Flowchart of subject selection process for pre-Hajj and post-Hajj among Indonesian pilgrim in 2015.

In 2015, Indonesia sent 168,800 pilgrims to Saudi Arabia [3]. The pilgrims embarked from 12 departure points (cities) around Indonesia and arrived in Indonesia post-Hajj at the same points. Each departure points consisted of 20–60 groups, of each consisted of 300–450 pilgrims. From all 12 departure points, five departure points (N = 90,998 pilgrims aged >18 years old) were selected for this study to represent west, central, and east regions of Indonesia. The selected regions consisted of three small departure points i.e.: (Banda Aceh/Medan), (Banjarmasin/Palangkaraya), and (Makassar/Gorontalo), and two large departure points i.e.: (Jakarta/Bekasi) and Surabaya. We randomly selected five departure groups from small departure points, and 10 departure groups from large departure points with total potentially eligible pilgrims are 12,455 (in total 35 departure groups) (Fig 1). Study subjects were randomly selected from each of 35 departure groups.

### Data collection

We conducted the data collection from September until October 2015. Average duration of travel for pilgrims was 40 days, consisted of 22 days in Mecca, 5–6 days in Arafah-Mina, 8–9 days in Medinah and 1–2 days in transit in Jeddah [5]. Pre-Hajj data collection was conducted at Hajj dormitories 24 hours prior to departure time. Informed consent forms were obtained before collecting the data. Several demographics characteristics (age, gender, education, socio-economic status), clinical data (comorbidity, symptoms including fever, sore throat, cough, shortness of breath, hospitalization, and antibiotic use), smoking status, and previous vaccination status were collected and recorded in the case report form. The Post-Hajj data collection, including clinical data (hospitalization, antibiotic use, and symptoms including fever, sore throat, cough, shortness of breath), was conducted immediately upon arrival from the same subjects when they arrived at the airport or in transit at the Hajj dormitory in Indonesia. The participants were identified with various methods in each departure points to minimize the risk of loss to follow up. The local authorities responsible for the arrival process, helped to identify the participants either by giving announcements when they move from the airport to the dormitory, by using a signboard, or through announcements in the Hajj dormitory.

### Sample collection

Nasopharyngeal (NP) swab specimens were collected in pre-Hajj and post-Hajj by trained medical personnel using a flexible nasopharyngeal flocked swab (Copan, Italy no 503SC01). The specimens were immediately placed into 1 mL of Skim milk Tryptone Glucose and Glycerol (STGG). The NP-STGG was vortexed and kept at 4°C for maximum 4–6 hours after collection [10]. The specimens were then shipped to Eijkman Institute for Molecular Biology in Jakarta and stored at -80°C until further analysis. The detail protocols were available at protocol.io (dx.doi.org/10.17504/protocols.io.bkuakwse).

### Isolation and Identification of *S*. *pneumoniae*

A 20 μl of NP-STGG was transferred and streaked onto 8% sheep blood agar plate supplemented with 5 mg/L gentamicin (SB-Gent) then incubated at 37°C with 5% CO_2_ for 18–24 h [11, 12]. Alpha-hemolytic and flat-depressed colonies resembling pneumococci were tested for the susceptibility to optochin (Oxoid) and bile solubility. Isolates showing susceptibility to optochin (a zone of inhibition of 14 mm or greater) and bile-soluble were confirmed as *S*. *pneumoniae*. These isolates were stored in STGG at -80°C until further analysis [13].

### DNA extraction

The DNA extraction of *S*. *pneumoniae* was performed by boiling method. Stocked bacteria in STGG were streaked onto 8% sheep blood agar plate then incubated at 37°C with 5% CO_2_ for 18–24 h. Fresh culture was harvested into 300 μl TE buffer in 1.5 mL microcentrifuge tube and vortexed. Bacterial suspension was heated at 100°C for 5 minutes and immediately placed in -20°C for 5 minutes then centrifuged at 13,000 × g for 10 minutes [14].

### Serotyping of *S*. *pneumoniae*

The serotyping was performed by sequential multiplex PCR (smPCR) targeting *wzy* gene, which encodes oligosaccharide repeat unit polymerases that specific among serotypes. The smPCR serotyping was consisted of 8 reactions that covered 70 serotypes as described by Centers for Disease Control and Prevention [14, 15]. Mastermix was prepared as follows, 1 × PCR buffer (Promega), 3.5mM of MgCl_2_, 200μM of dNTPs (Promega), varied concentration of primer pairs ranging from 0,1μM to 0,5μM, 2U of *Taq* DNA Polymerase and 2.5 μl of DNA template. Nuclease-free water was added to final volume 25 μl. The PCR was run under following condition: pre-denaturation at 94°C for 4min, followed by 30 cycles of denaturation at 94°C for 45sec, annealing at 54°C for 45sec, and elongation at 65°C for 2min 30sec. The PCR products were visualized by gel electrophoresis using 2% agarose gel at 100V for 90 minutes. The non-typeable isolates were confirmed by real-time PCR targeting *lytA* to confirm that the isolate was *S*. *pneumoniae*. Isolate showed positive results for *lytA* was assigned as non-typeable (NT) by PCR [14, 15].

### Antimicrobial susceptibility testing

Antimicrobial susceptibility testing of *S*. *pneumoniae* was performed by following Clinical and Laboratory Standards Institute (CLSI) guideline 2017. Pure and freshly grown culture was added into 5 mL saline 0.85% in glass tube then the turbidity was adjusted to 0.5 McFarland standard. Sterile cotton swab was dipped into suspension and pressed gently onto the wall of glass tube to remove excess fluid. The cotton swab was lawn onto Mueller Hinton Agar (MHA) plate with 5% sheep blood and the plate was lawn 3 times by rotating in 60° direction. Antibiotic disks (Oxoid) were placed onto the agar. Clindamycin 2 μg, chloramphenicol 30 μg, oxacillin 1 μg, tetracycline 30 μg, erythromycin 15 μg, and trimethoprim-sulfamethoxazole (co-trimoxazole) 1,25/23.75 μg, were used in this study. Oxacillin disk was applied to measure susceptibility of isolates to penicillin. Inoculated media were incubated in 37°C with 5% CO_2_ for 20–24 h. Inhibition zone was measured and recorded. Susceptibility was determined according to breakpoints in CLSI guideline 2017 [16].

### Sample size

According to study by Farida H, et al., the prevalence of *S*. *pneumoniae* was reported in 11% of population [17]. Study by Memish et.al conducted in 2011 and 2012 hajj seasons enrolling adult pilgrims from 18 countries in Africa or Asia, found a statistical significant increase in carriage between beginning-Hajj and end-Hajj cohorts for overall carriage from 4.4% versus 7.5%, prevalence ratio [PR] = 1.7, 95% CI = 39 1.3–2.3) [9].

Based on these studies, we estimated the proportion of carriage post-Hajj will be 15%, hence the sample size from each selected departure points/embarkation can be determined according to sample size formula if population size is known, with 95% confidence level and half-width precision of 5%, resulting of minimum sample size at least 225 subjects for each departure points.

### Data analysis

All the subjects who had provided pre- and post-Hajj NP swab samples were accounted in the analysis. Characteristics of subjects were summarized as counts and percentages.The prevalence of pneumococcal carriage pre- and post-Hajj was compared with the McNemar test and reported in proportion with 95% confidence interval around the estimate. The measures were also reported for each region. A *p value* < 0.05 was considered significant.

## Results

### Demographics and clinical characteristics of Indonesian Hajj pilgrims in 2015

A total of 1153 subjects were enrolled in this study at pre-Hajj (Fig 1). Upon the return for post-Hajj data collection, 247 participants were lost to follow-up due to not showing up during data collection; 50 participants were lost to follow up due to sickness, or decline to participate; 7 participants died during the Minas’ Tragedy, and 34 specimens were not available. Additionally, 2 subjects did not have pre-hajj specimens available (Fig 1). Therefore, final data analysis consists a total of 813 subjects with both pre-Hajj and post-Hajj data available. The baseline characteristic of these subjects is described in Table 1. The mean of age of the participants was 53.1 years old, with 68.5% aged 18–59 years old and 27.6% above 60 years old. About half of them were male (50.6%), most of the subjects were high-school (37.6%) or university educated (25.7%), and 15.5% of participants were active smoker (Table 1). Half of the subjects were from west region of Indonesia including Sumatra and Java (51.9%), followed by central region (Kalimantan, Bali, Nusa Tenggara) (24.7%) and east region (Sulawesi) (23.4%). The hajj participants majority lived with 4 to 6 family members (58.5%), followed by 1 to 3 family members (35%) and 7 to 9 family members (7%). 114 (14.0%) participants lived with children under five years old. In this study, 167 participants (20.5%) had history of comorbidity and only 18 (2.2%) participants had a history of pneumococcal vaccination (Table 1).

**Table 1:** Characteristics of Indonesian Hajj pilgrims in 2015 (n = 813).

Variables		n (%)
Age category (years)		
	18 to <60	557 (68.5)
	≥ 60	224 (27.6)
Gender		
	Male	411 (50.6)
Region		
	West Indonesia	422 (51.9)
	Central Indonesia	201 (24.7)
	East Indonesia	190 (23.4)
Active smoker		126 (15.5)
Education background		
	None	96 (11.8)
	Primary school	201 (24.7)
	High school	306 (37.6)
	University	209 (25.7)
Activity		
	Housewife	218 (26.8)
	Worker	180 (22.1)
	Entrepreneur	206 (25.3)
	Retirement	56 (6.9)
	Farmers	45 (5.5)
	Others	107 (13.2)
Number of cohabitants		
	1–3	277 (34.1)
	4–6	476 (58.5)
	≥7	60 (7.4)
Living with children <5 years		114 (14.0)
Comorbidities		167 (20.5)
	Chronic obstructive pulmonary disease	6 (0.7)
	Lung tuberculosis	7 (0.9)
	Bronchiectasis	3 (0.4)
	Heart failure	6 (0.7)
	Chronic liver disease/Liver cirrhosis	2 (0.2)
	Stroke	1 (0.1)
	Diabetes mellitus	47 (5.8)
	Other comorbidities	116 (14.3)
History of pneumococcal vaccine		18 (2.2)

The clinical characteristics of subjects before and during Hajj are described in Table 2. The number of pilgrims with one or more respiratory symptoms were increased, from 44.5% in pre-Hajj to 85.6% during Hajj, as well as those that require antibiotic therapy in previous three months, from 8% in pre Hajj to 47.8% during Hajj. We reported that 9 subjects were hospitalized during the Hajj, meanwhile 21 of 813 subjects have reported referred to hospital during three months before departure.

**Table 2:** Clinical characteristics of paired Indonesian Hajj pilgrims in 2015 (n = 813).

Variables	Pre-Hajj (N = 813)	During Hajj (N = 813)
Respiratory symptoms***	362 (44.5%)	696 (85.6%)
Productive cough	139 (17.1)	532 (65.4)
Dry cough	166 (20.4)	231 (28.4)
Fever	141 (17.3)	214 (26.3)
Sore throat	116 (14.3)	280 (34.4)
Cold	196 (24.1)	420 (51.7)
Ear pain	19 (2.3)	4 (0.5)
Hospital admission*	21 (2.6)	9 (1.1)
Antibiotic use**	65 (8.0)	389 (47.8)

*in the last 1 year for pre-Hajj.

**in the last 3 months.

*** one or more respiratory symptoms.

### Prevalence and serotype distribution of *S*. *pneumoniae* carriage

We observed that the pre-Hajj prevalence of pneumococcal carriage among 813 of Indonesian Hajj pilgrims in 2015 were 8.6% (95% CI 6.7–10.5%) (n = 70, Table 3). We found the prevalence of *S*. *pneumoniae* in pre-Hajj was higher in subjects from East (18.9%), followed by west (5.5%) and central (5.5%) of Indonesia (Table 3).

**Table 3:** Prevalence of *S*. *pneumoniae* carriage among Indonesian Hajj before departure to Saudi (Pre-Hajj) and upon arrival (Post-Hajj) in different regions in Indonesia 2015.

Region	Islands	N	Pre-Hajj		Post-Hajj		*p* value
			n	%	n	%	
All		813	70	8.6	67	8.2	0.844
West	Sumatera, Java	422	23	5.5	28	6.6	0.551
Central	Kalimantan, Bali, Nusa Tenggara	201	11	5.5	18	9.0	0.210
East	Sulawesi	190	36	18.9	21	11.1	0.018

The pneumococcal carriage was 8.2% after the Hajj (95% CI 6.4–10.1%) (n = 67, Table 3). Among the 67 subjects with pneumococcal carriage, 50 of them were acquired during Hajj. Compared with pre-Hajj, the prevalence increased in subject from west (from 5.5% to 6.6%; *p value*: 0.551) and central (from 5.5% to 9.0%; *p value*: 0.210) regions but decreased in subject from east region (from 18.9% to 11.1%; *p value*: 0.018) (Table 3).

**Fig 2:**
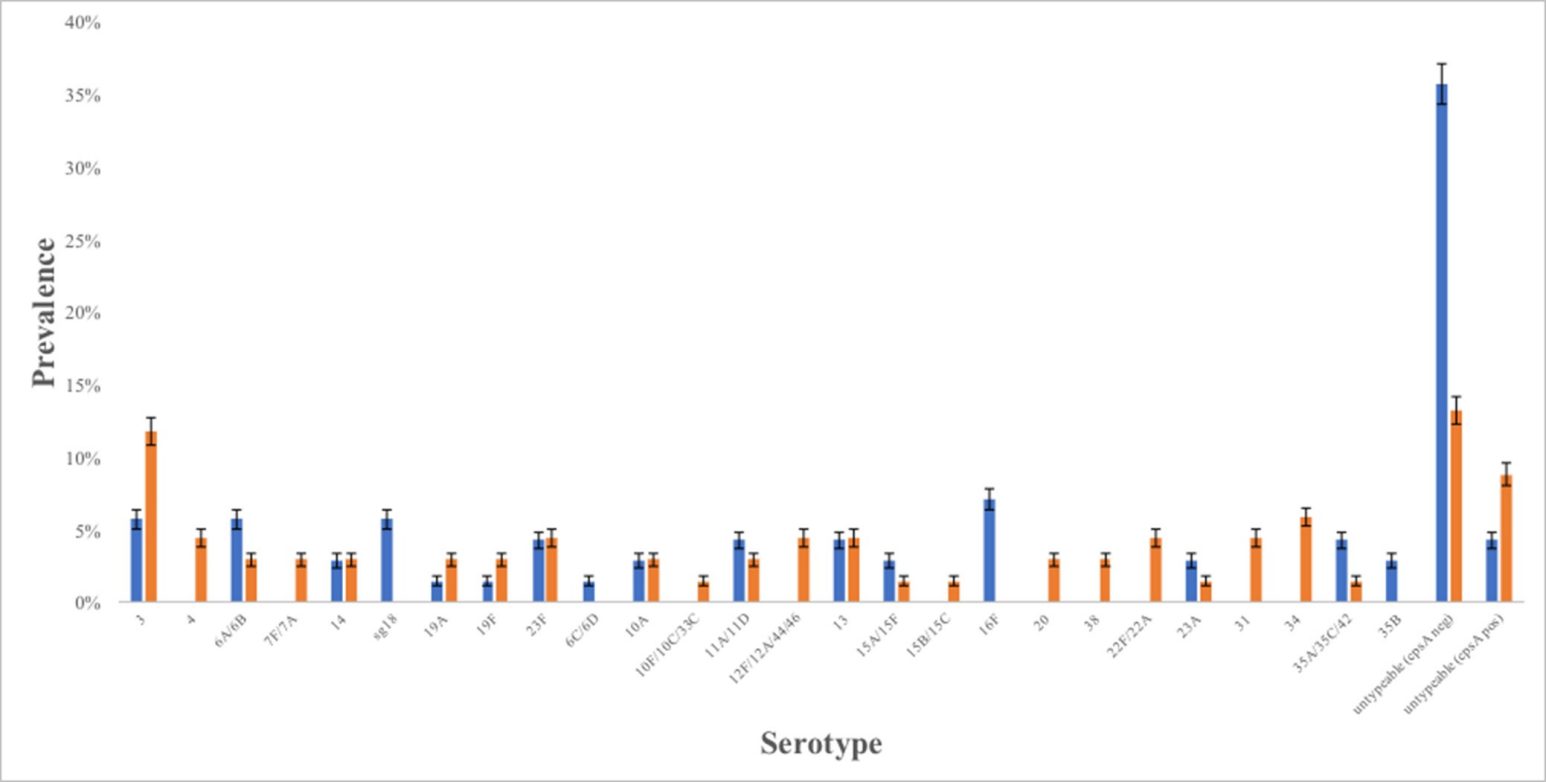
Serotype distribution of *Streptococcus pneumoniae* carriage pre-Hajj (n = 70, blue bar) and post-Hajj (n = 68, orange bar) among Indonesian Hajj pilgrims in 2015. Error bars represent 95%CI.

All of the 70 pre-Hajj and 67 post-Hajj *S*. *pneumoniae* strains were cultured for serotyping. The most common serotype observed among pre-Hajj samples was serotype 16F (n = 5; 7.1%) followed by 6A/6B, 3, and 18 (n = 4 (5.7%) each), 23F, 11A/11D, 13, and 35A/35C/42 (n = 3 (4.3%) each), 14, 10A, 15A/15F, 23A, and 35B (n = 2 (2.9%) each), 19A, 19F, and 6C/6D (n = 1(1.4%) each) (Fig 2; green bar). Meanwhile, the most commonly observed among post-Hajj samples was serotype 3 (n = 8; 11.8%) followed by 34 (n = 4; 5.9%), 4, 23F, 12F/12A/44/46, 22F/22A, 13, and 31(n = 3 (4.4%) each), 6A/6B, 7F/7A, 14, 19A, 19F, 10A, 11A/11D, 20, and 38 (n = 2 (2.9%) each), 15B/15C, 10F/10C/33C, 1A/15F, 23A, and 35A/35C/42 (n = 1 (1.5%) each) (Fig 2; red bar). We found that 28 (40.0%) isolates in pre-Hajj and 15 (22.1%) isolates in post-Hajj were untypeable (NT) using the smPCR method, with 25 out of 28 isolates (14% of all; pre-Hajj samples) and 9 out of 15 (9% of all; post-Hajj samples) also being PCR-negative for the *cpsA* gene.

In this study, strains isolated from pre-Hajj samples that could be covered by the pneumococcal vaccine varied between 27.1% to 28.6% for PCV13 and PPV23 vaccines, respectively. For post-Hajj samples, strains that could be covered by the pneumococcal vaccine varied between 35.3% to 51.5% for PCV13 and PPV23 vaccines, respectively.

### Antibiotics susceptibility profile of *S*. *pneumoniae* strains

We observed that the majority of strains obtained from pre-Hajj samples (n = 70 isolates) were susceptible to erythromycin (88.6%), clindamycin (87.1%), chloramphenicol (84.3%), penicillin (50.0%), co-trimoxazole (42.9%) and tetracycline (41.4%). Meanwhile, the strains obtained from post-Hajj (n = 68 isolates) were susceptible to clindamycin (88.2%), erythromycin (79.4%), chloramphenicol (76.5%), co-trimoxazole (57.4%), penicillin (51.5%), and tetracycline (35.3%) (Table 4). We found that the antibiotic susceptibility to co-trimoxazole agent is higher in post-hajj strains compared to pre-Hajj, meanwhile the tetracycline and chloramphenicol susceptibility were lower in post-Hajj- compare to pre-Hajj (Table 4).

**Table 4:** Antimicrobial susceptibility of *Streptococcus pneumoniae* strains carried by Indonesian pilgrims pre- and post-Hajj in 2015.

Antimicrobial agent	Number (%) of susceptible isolates	
	Pre-hajj (n = 70)	Post-hajj (n = 68)
Chloramphenicol	59 (84.3)	52 (76.5)
Erythromycin	62 (88.6)	54 (79.4)
Clindamycin	61 (87.1)	60 (88.2)
Tetracycline	29 (41.4)	24 (35.3)
Co-trimoxazole	30 (42.9)	39 (57.4)
Penicillin*	35 (50.0)	35 (51.5)

*Susceptibility to penicillin was determined with oxacillin disk.

## Discussion

This is the first multisite longitudinal study to determine the prevalence of pneumococcal carriage in Indonesia, the serotypes distribution of *S*. *pneumoniae* carriage and antibiotic susceptibility of *S*. *pneumoniae* isolated from Indonesian Hajj pilgrims before and after Hajj. In general, this study showed no difference in pneumococcal carriage prevalence between pre-Hajj (8.6%) and post-Hajj (8.2%). Furthermore, we observed that the prevalence of *S*. *pneumoniae* carriage in pre-Hajj was higher in east region than west and central regions of Indonesia. The prevalence of *S*. *pneumoniae* carriage in western of Indonesia was also reported low in elderly subjects. A previous study conducted in Jakarta reported that prevalence of elderly subjects with *S*. *pneumoniae* colonization in nasopharynx was only 2,6% [18]. Meanwhile in Eastern of Indonesia, there is no report about the prevalence of *S*. *pneumoniae* in the elderly, but there were other studies that mentioned prevalence of *S*. *pneumoniae* carriage in children living in Lombok. The reports mentioned that half of the children (50% and 46%) in Lombok carried *S*. *pneumoniae* in nasopharynx [11, 19]. This difference is caused by many factors, some of the factors are the number of urban and rural areas between Western and Eastern Indonesia. There are more rural areas in Eastern Indonesia which affects sanitation and higher prevalence of *S*. *pneumoniae* colonization. A study reported that rural and urban status of a region were one of risk factors affecting *S*. *pneumoniae* and other bacteria colonization in nasopharynx [20].

Meanwhile, the prevalence of *S*. *pneumoniae* carriage in post-Hajj increased among the subjects from west and central regions but decreased in the east region. Previous studies also reported that there were acquisition of *S*. *pneumoniae* nasal carriage in Hajj pilgrims returned from Saudi Arabia among French pilgrims in the 2012 resulting an increase of pneumococcal carriage prevalence in post-Hajj [8]. Another prospective cohort study of 129 pilgrims who departed from France in 2013 confirms a high acquisition of rhinovirus and *S*. *pneumoniae* in pilgrims. *S*. *pneumoniae* were positive in 50.0% and 62.0% of pre-Hajj and post-Hajj specimens, respectively. During their stay, one-third (36.3%) of the participants had acquired *S*. *pneumoniae* [1]. The reduction of colonization in subjects from eastern regions could be caused by the high number of prevalence pre-Hajj. This finding describes that the pilgrimage hajj might affect *S*. *pneumoniae* colonization by increasing or decreasing the prevalence of *S*. *pneumoniae* carriage rate. This decreasing was also reported in previous study conducted in some countries in Africa and Asia where country with high prevalence in pre-hajj showed decreasing of *S*. *pneumoniae* colonization prevalence in post hajj, such as India (6.8% to 6.1%), which was also concordant with decreasing of *S*. *pneumoniae* colonization prevalence in east region of Indonesia (18.9% to 11.1%) [9].

In a paired cohort study of *S*. *pneumoniae* in 2013 observed 12% acquisition rate. The nasal carriage rate was 5.6% at the beginning of the Hajj and 12.7% at the end of the Hajj [21]. Another study prospective longitudinal-cohort study was done in Saudi Arabia in 2013 found that the overall carriage rate of *S*. *pneumoniae* in the pre- and post-Hajj was 1.8% and 7.1% (*p*-value = 0.0016) [22]. In 2016, a multi-site longitudinal surveillance study from 807 Indian pilgrims detected a non-significant increase of carriage rate between pre- and post-Hajj cohorts: Nasopharyngeal carriage- 5.6% versus 7.8%, and oropharyngeal carriage- 7.4% versus 8.6%, respectively (*p*-value = 0.09) [22]. Nonetheless, the study confirmed high acquisition rate of multidrug-resistant *S*. *pneumoniae*, from 11% in pre-Hajj group to 32% in post Hajj group (*p*-value = 0.0002) [22]. The increase in *S*. *pneumoniae* transmission was caused by interaction with residents during the Hajj. This transmission was amplified in crowded area that incidences triggered transmission such as sneezing, and coughing might be found more often [9].

Pneumococcal serotype prevalence varied widely between and within countries [23]. In terms of *S*. *pneumoniae* colonization, our study showed an acquisition of serotype 3 in Indonesian Hajj Pilgrims [24]. This serotype remains a major cause of invasive pneumococcal disease. Although Indonesia has limited published data on serotype prevalence, two nasopharyngeal carriage studies provided serotype distribution in healthy adults. One study detected four serotypes (3, 6A/B, 15B/C, 35F) among 16 pneumococcal isolates collected from 149 adults aged 60 to 97 years in Jakarta [12]. Another study of 253 adults aged 45 to 70 years in Semarang reported that serotype 6A/B was most common (39% of pneumococcal isolates) followed by 15B/C, and 15A [17]. In this study, we recognized there was an increase of vaccine serotypes between pre-Hajj and post-Hajj from 29% (20/70) to 53% (36/67). We found that acquired serotypes, such as serotype 4, 7F/7A, 12F/12A/44/46,15B/15C, 20 and 22F/22A and increasing numbers of colonization for serotype 3, 19F and 19A, were included in pneumococcal vaccine (PCV13 and PPSV23). According to previous studies about serotype circulating in Saudi Arabia, some of acquired serotypes are included in dominant serotypes (4, 3, 19F, 9V, 6A, 19A, 14, 23F) and less common serotypes (6B, 7A, 17F, 22F, 15B, 23B, 9N, 19C, and 20) circulating in Saudi Arabia [25, 26]. While some serotypes were no longer detected in post-Hajj, i.e. serotype 18 (Fig 2). The serotype distribution of our study is quite similar with the Memish et al. that found serotype 3 were the most prevalent post-Hajj [9]. The subgroup analysis of the new acquired pneumococcal carriage also found that serotype 3 is the most prevalent serotype in our study. Meanwhile, other different prevalent serotypes were also identified in the Indian pilgrims’ study, such as serotype 19F (20%), 9V (7.2%), 1 (6.2%), and 31 (6%) during post Hajj [27]. These vaccine serotypes circulating in Saudi Arabia might cause invasive pneumococcal diseases incidences during Hajj.

Although we found a difference proportion in antibiotic use in Saudi Arabia between the carriage and non-carriage group, the results were heavily relied on the self-reported data from study subjects. Data on antibiotic use were collected by interviewing the subjects upon arrival at the Hajj dormitory to recall their history of antibiotic use in Saudi Arabia. They may not be able to clearly identify whether they took antibiotics or any other medications. Thus, non-differential misclassification of antibiotic uses may lead to overestimation of the number of antibiotic use in both carriage and non-carriage groups. The Indian pilgrims study suggested the increased carriage of antibiotic-resistant pneumococci in the post-Hajj cohort may be associated with self-medication and excessive use of antibiotics [27]. Regarding to antibiotic use, in this study, we found that acquired *S*. *pneumoniae* in post-Hajj showed less susceptibility compared to pre-Hajj. However, the non-susceptibility pattern in post-Hajj is still similar with pre-Hajj with tetracycline, penicillin and co-trimoxazole are detected to be less susceptible. A study about antibiotic resistance pattern of *S*. *pneumoniae* in Saudi Arabia reported that *S*. *pneumoniae* showed high resistant to erythromycin, penicillin, clarithromycin and cefuroxime [26].

In terms of substantiation, this is the first study described the prevalence of *S*. *pneumoniae* carriage, serotypes distribution and antibiotic susceptibility profile before and after Hajj within the same subjects in Indonesia. Regarding the method, the strength of this study is the application of a stratified cluster random sampling to minimize the selection bias and several preventive measures were taken to minimize the number of drop-out. Limitation of the study is the significant number of subjects who were dropped out after Hajj. To analyze the internal validity, we performed a missing value analysis to compare characteristic of the subjects who were dropped out with those who completed the study. Our analysis revealed that the dropped-out subjects were comparable with the ones who completed the study in terms of mean age, age categories, gender, antibiotic use in the past three months, number of co-habitants, smoking status, and history of comorbid diseases. Therefore, it is less likely that the proportion of dropped out participants will affect the validity of this study.

In conclusion, our study revealed the acquisition of some vaccine serotypes (PCV13 and PPSV23) in Indonesian Hajj pilgrims nasopharynx. These findings might suggest the potential role of pneumococcal vaccine before departure to reduce nasopharyngeal colonization and invasive pneumococcal diseases during Hajj pilgrimage.

## Supporting information

S1 File Questionnaire forms. (PDF)
