# Supplementary material for: Streptococcus pneumoniae carriage and antibiotic susceptibility among Indonesian pilgrims during the Hajj pilgrimage in 2015
Source: PLoS One. 2021 Jan 26;16(1):e0246122. doi: 10.1371/journal.pone.0246122 (PMC7837496; doi:10.1371/journal.pone.0246122)
Supplement: S1 File — (PDF) [file pone.0246122.s001.pdf]

Case Report Form - "Molecular Epidemiology of Streptococcus pneumoniae in Indonesian Hajj"

Unit CEEBM CMH-FMUI, Eijkman Molecular Biology Institute, and Hajj Health Center Indonesia MoH
Principal Investigator: Dr. dr. Kuntjoro Harimurti, SpPD-KGER, MSc

Stick the barcode inside this box

A. Data Source Information

A1. Enumerator Name: ....

A2. Sample collecting date: dd ☐☐ - Sept- 2015

A3. Embarkation: 1○ Banda Aceh 4○ Makassar/Gorontalo; 2○ Medan; 5○ Banjarmasin/Palangkaraya; 3○ Jakarta/Bekasi; 6○ Surabaya

A4. Flight group number / Kloter : ☐☐☐ e.g.: 023

B. Demography

B1. Date of birth: dd ☐☐ - mm ☐☐ - yyyy ☐☐☐☐ or 99○ Unknown;

B2. Gender: 1○ Male; 2○ Female;

B3. Completed education:
1○ None; 4○ Senior high school;
2○ Primary school; 5○ College
3○ Junior high school; 6○ University

B4. Living place status:
1○ Self-owned; 5○ Office's;
2○ Parents'/child's; 6○ Orphanage;
3○ Relative's; 7○ Others, i.e.:
4○ Rent; ....

B5. House area ☐☐☐☐ m2
or 99○ Unknown;

B6. Activity:
1○ Unemployed; 6○ Retired govn't officer;
2○ HouseweIf answer toe; 7○ Retired private employee;
3○ Government officer; 8○ Others, i.e.:
4○ Private employee ....
5○ Entrepeneur;

B7. Total family member/ person living in the house (including study subject): ☐☐ people
with detail:

B7a. Total elderly (age ≥ 60 years) ☐☐ people

B7b. Total adults (18 ≤ age < 60 years) ☐☐ people

B7c. Total children (5 ≤ age < 18 years): ☐☐ people

B7d. Total children (2 ≤ age < 5 years): ☐☐ people

B7e. Total children (age < 2 years): ☐☐ people

C. Clinical data

C1. In the last 1 year, often have the following symptoms:

	C1a. Symptoms			C1b. If answer to answer to C1a (yes), how often in a month?		
**1.**	Phlegm	0○ No	1○ Yes	1○ <once	2○ once	3○ >once
**2.**	Dry cough	0○ No	1○ Yes	1○ <once	2○ once	3○ >once
**3.**	Fever	0○ No	1○ Yes	1○ <once	2○ once	3○ >once
**4.**	Sore throat	0○ No	1○ Yes	1○ <once	2○ once	3○ >once
**5.**	Flu	0○ No	1○ Yes	1○ <once	2○ once	3○ >once
**6.**	Earache	0○ No	1○ Yes	1○ <once	2○ once	3○ >once

Untuk setiap pertanyaan:
Beri tanda (✓) pada ○ yang sesuai; atau Isi dengan angka pada ☐; atau Tulis jawaban pada ....

Case Report Form - "Molecular Epidemiology of Streptococcus pneumoniae in Indonesian Hajj"

Unit CEEBM CMH-FMUI, Eijkman Molecular Biology Institute, and Hajj Health Center Indonesia MoH

Principal Investigator: Dr. dr. Kuntjoro Harimurti, SpPD-KGER, MSc

C2. Hospital admission in last 3 months: 0O No (continue to no C3) 1O Yes

C2a. If answer to answer to C2 (Yes), duration of admission: ☐☐☐ days 99O Unknown

C3. Antibiotic use in the past 3 months: 0O No 1O Yes 99O Unknown

C4. History of comorbid disease: 0O None (continue to no C5) 1O Yes, If answer to C4 (Yes), please mention:

**C4a.**	COPD	0O None	1O Yes
**C4b.**	Lung TB	0O None	1O Yes
**C4c.**	Bronchiectasis	0O None	1O Yes
**C4d.**	Heart failure	0O None	1O Yes
**C4e.**	Chronic liver disease/ liver chirrosis	0O None	1O Yes
**C4f.**	Stroke	0O None	1O Yes
**C4i.**	Diabetes melitus	0O None	1O Yes
**C4k.**	Others, i.e.: …………………………………………….	0O None	1O Yes

C5. In the last 5 years, subject has received pneumococcal vaccine before: 0O No (continue to no C6) 1O Yes

If answer to C5 (Yes):

C5a. Last vaccination in year: ☐☐☐☐ 99O Unknown

C5b. Vaccine type: 1O PCV 13 2O PPV23 3O PCV 7 4O PCV10 99O Unknown

C6. Subject is smoker: 0O No (continue to no C7) 1O Yes 2O Yes before but now already stop

C6a. If answer to C6 (yes), total cigarrete per day: ☐☐☐ cigarrete per day

If answer to C6 (Yes before but now already stop):

C6b. Stop in year: ☐☐☐☐; **C6c.** When smoking before, total cigarette per day: ☐☐☐ cigarrete per day

C7. Family living in the same house who smoke: 0O No 1O Yes, if answer to C7 (Yes)

	**C7a.** Family relationship with subject	**C7b.** Total cigarrete per day?
1.	1O Parents; 2O Husband/wife; 3O Child; 4O Sibling; 5O Others, i.e.:…………………….;	☐☐☐ cigarrete per day; 99O Unknown
2.	1O Parents; 2O Husband/wife; 3O Child; 4O Sibling; 5O Others, i.e.:…………………….;	☐☐☐ cigarrete per day; 99O Unknown
3.	1O Parents; 2O Husband/wife; 3O Child; 4O Sibling; 5O Others, i.e.:…………………….;	☐☐☐ cigarrete per day; 99O Unknown
4.	1O Parents; 2O Husband/wife; 3O Child; 4O Sibling; 5O Others, i.e.:…………………….;	☐☐☐ cigarrete per day; 99O Unknown

Completed by:

Personnel name : ……………………………………………… Signature: ☐

Date : dd☐☐ - September - 2015

For every question:

Please tick (✓) in suitable O; or fill in with numbers in ☐; or write answer in………

Case Report Form - "Molecular Epidemiology of Streptococcus pneumoniae in Indonesian Hajj"

Unit CEEBM CMH-FMUI, Eijkman Molecular Biology Institute, and Hajj Health Center Indonesia MoH

Principal Investigator: Dr. dr. Kuntjoro Harimurti, SpPD-KGER, MSc

Stick the barcode inside this box

E. A. Data Source Information

E1. Enumerator Name: ....

E2. Sample collecting date: dd☐☐ - Oct- 2015

E3. Embarkation: 1○ Banda Aceh 2○ Medan; 3○ Jakarta/Bekasi; 4○ Makassar/Gorontalo; 5○ Banjarmasin/Palangkaraya; 6○ Surabaya

E4. Flight group number / Kloter : ☐☐☐ e.g.: 023

F. Exposure during Hajj Pilgrimage

F1. Hospital admission in Saudi Arabia: 0○ No (continue to no F2) 1○ Yes

F1a. If the answer to F1 (Yes), admission duration: ☐☐☐ days 99○ Unknown

F1b. If the answer to F1 (Yes), based on indication: .... 99○ Unknown

F2. Experiencing following symptoms while in Saudi Arabia:

	**F2a.** Symptoms			**F2b.** If answer to answer to F2a (yes), how often while in Saudi Arabia ?		
**1.**	Phlegm	0○ No	1○ Yes	1○ <once	2○ once	3○ >once
**2.**	Dry cough	0○ No	1○ Yes	1○ <once	2○ once	3○ >once
**3.**	Fever	0○ No	1○ Yes	1○ <once	2○ once	3○ >once
**4.**	Sore throat	0○ No	1○ Yes	1○ <once	2○ once	3○ >once
**5.**	Flu	0○ No	1○ Yes	1○ <once	2○ once	3○ >once
**6.**	Earache	0○ No	1○ Yes	1○ <once	2○ once	3○ >once

F3. During your stay in Saudi Arabia, have you ever stay with minimal 1 person who have respiratory tract/lung infection?

0○ No (continue to no F4) 1○ Yes

It is said Respiratory tract infection if there is symptoms of cough,flu,sorethroat, hurt when swallow, with or without fever

It is said lung infection if there is productive cough (phlegm), difficulty breathing, pleuritic chest pain, with or without fever

F4. The use of antibiotic during stay in Saudi Arabia: 0○ No 1○ Yes 99○ Unknown

Completed by:

Personnel name : .... Signature:

Date : dd☐☐ - October - 2015

For every question:

Please tick (✓) in suitable ○; or fill in with numbers in ☐; or write answer in.........

Versi 5.0; date: 9 October 2015

Case Report Form - "Epidemiologi Molekular *S. pneumoniae* pada Jemaah Haji Indonesia"

Unit CEEBM RSCM-FKUI, Lembaga Biologi Molekular Eijkman, dan Pusat Kesehatan Haji Kementerian Kesehatan RI
Peneliti utama: Dr. dr. Kuntjoro Harimurti, SpPD-KGER, MSc

Stick the barcode inside this box

A. Informasi Sumber Data

A1. Nama Enumerator: ....

A2. Tanggal pengambilan sampel: dd☐☐ - Sept- 2015

A3. Embarkasi: 1O Banda Aceh 4O Makassar/Gorontalo; 2O Medan; 5O Banjarmasin/Palangkaraya; 3O Jakarta/Bekasi; 6O Surabaya

A4. Nomor Kelompok terbang / Kloter : ☐☐☐ contoh: 023

B. Demografi

B1. Tanggal lahir: dd☐☐ - mm☐☐ - yyyy☐☐☐☐ atau 99O Tidak diketahui;

B2. Jenis kelamin: 1O Laki-laki; 2O Perempuan;

B3. Pendidikan terakhir (yang ditamatkan):
1O Tidak sekolah; 4O SMA;
2O SD; 5O Akademi
3O SMP; 6O Perguruan Tinggi

B4. Status tempat tinggal:
1O Rumah sendiri; 5O Rumah dinas;
2O Rumah orang tua/anak; 6O Panti/yayasan;
3O Rumah saudara; 7O Lain-lain, sebutkan:
4O Rumah kontrakan; ....

B5. Luas rumah (bangunan) ☐☐☐☐ m2
atau 99O Tidak diketahui;

B6. Aktivitas:
1O Tidak bekerja; 6O Pensiun PNS;
2O Ibu rumah tangga; 7O Pensiun Swasta;
3O Karyawan PNS; 8O Lain-lain, sebutkan:
4O Karyawan Swasta
5O Wiraswasta; ....

B7. Jumlah total anggota keluarga / orang yang tinggal serumah (termasuk subjek penelitian): ☐☐ orang

Dengan rincian:

B7a. Jumlah orang tua (usia ≥ 60 tahun) ☐☐ orang

B7b. Jumlah orang dewasa (18 ≤ usia < 60 tahun) ☐☐ orang

B7c. Jumlah anak-anak usia (5 ≤ usia < 18 tahun): ☐☐ orang

B7d. Jumlah anak-anak usia (2 ≤ usia < 5 tahun): ☐☐ orang

B7e. Jumlah anak-anak (usia < 2 tahun): ☐☐ orang

C. Data Klinis

C1. Dalam 1 tahun terakhir, sering mengalami gejala penyakit berikut:

	**C1a.** Gejala penyakit			**C1b.** Jika jawaban D2a (ya), seberapa sering terjadi dalam 1 bulan?		
**1.**	Batuk berdahak	0O Tidak	1O Ya	1O <1 kali	2O 1 kali	3O >1 kali
**2.**	Batuk kering	0O Tidak	1O Ya	1O <1 kali	2O 1 kali	3O >1 kali
**3.**	Demam	0O Tidak	1O Ya	1O <1 kali	2O 1 kali	3O >1 kali
**4.**	Sakit tenggorokan	0O Tidak	1O Ya	1O <1 kali	2O 1 kali	3O >1 kali
**5.**	Pilek	0O Tidak	1O Ya	1O <1 kali	2O 1 kali	3O >1 kali
**6.**	Sakit telinga	0O Tidak	1O Ya	1O <1 kali	2O 1 kali	3O >1 kali

C2. Perawatan RS dalam 3 bulan terakhir: 0○ Tidak (lanjutkan ke no C3) 1○ Ya

C2a. Bila jawaban C2 (ya), lama masa rawat: ☐☐☐ hari 99○ Tidak diketahui

C3. Penggunaan antibiotika dalam 3 bulan sebelumnya: 0○ Tidak 1○ Ya 99○ Tidak diketahui

C4. Riwayat penyakit komorbid: 0○ Tidak ada (lanjutkan ke no C5) 1○ Ada, Bila jawaban C4 (Ada), sebutkan:

**C4a.**	PPOK	0○ Tidak ada	1○ Ada
**C4b.**	Tuberkulosis paru	0○ Tidak ada	1○ Ada
**C4c.**	Bronkiektasis	0○ Tidak ada	1○ Ada
**C4d.**	Gagal jantung	0○ Tidak ada	1○ Ada
**C4e.**	Penyakit hati kronik/sirosis hati	0○ Tidak ada	1○ Ada
**C4f.**	Stroke	0○ Tidak ada	1○ Ada
**C4i.**	Kencing Manis (Diabetes melitus)	0○ Tidak ada	1○ Ada
**C4k.**	Lain-lain, sebutkan: …………………………………………….	0○ Tidak ada	1○ Ada

C5. Dalam 5 tahun terakhir, Subjek pernah menerima Vaksin pneumococcus sebelumnya: 0○ Tidak (lanjutkan ke no C6) 1○ Ya

Bila jawaban C5 (ya):

C5a. Vaksinasi terakhir kali pada tahun: ☐☐☐☐ 99○ Tidak diketahui

C5b. Jenis vaksin: 1○ PCV 13 2○ PPV23 3○ PCV 7 4○ PCV10 99○ Tidak diketahui

C6. Subjek merupakan perokok: 0○ Tidak (lanjutkan ke no C7) 1○ Ya 2○ Dulu YA, sudah berhenti

C6a. Bila jawaban C6 (ya), jumlah batang per-hari: ☐☐☐ batang

Bila jawaban C6 (Dulu YA dan sudah berhenti):

C6b. Berhenti pada tahun: ☐☐☐☐; **C6c.** Pada saat masih merokok, jumlah batang per-hari: ☐☐☐ batang

C7. Keluarga yang tinggal dalam satu rumah merokok: 0○ Tidak 1○ Ya, Bila jawaban C7 (ya)

**C7a.** Hubungan keluarga dengan subjek	**C7b.** Jumlah batang perhari?
1. 1○ Orang tua; 2○ Suami/Istri; 3○ Anak; 4○ Saudara; 5○ Lain-lain, sebutkan:……………………..;	☐☐☐ batang per-hari; 99○ Tidak diketahui
2. 1○ Orang tua; 2○ Suami/Istri; 3○ Anak; 4○ Saudara; 5○ Lain-lain, sebutkan:……………………..;	☐☐☐ batang per-hari; 99○ Tidak diketahui
3. 1○ Orang tua; 2○ Suami/Istri; 3○ Anak; 4○ Saudara; 5○ Lain-lain, sebutkan:……………………..;	☐☐☐ batang per-hari; 99○ Tidak diketahui
4. 1○ Orang tua; 2○ Suami/Istri; 3○ Anak; 4○ Saudara; 5○ Lain-lain, sebutkan:……………………..;	☐☐☐ batang per-hari; 99○ Tidak diketahui

Diisi oleh

Nama personnel : ………………………………………………… Tanda tangan: ☐

Tanggal : dd☐☐ - September - 2015

Case Report Form - "Epidemiologi Molekular *S. pneumoniae* pada Jemaah Haji Indonesia"

Unit CEEBM RSCM-FKUI, Lembaga Biologi Molekular Eijkman, dan Pusat Kesehatan Haji Kementerian Kesehatan RI
Peneliti utama: Dr. dr. Kuntjoro Harimurti, SpPD-KGER, MSc

Stick the barcode inside this box

E. Informasi Sumber Data

E1. Nama Personnel: ....

E2. Tanggal pengambilan sampel: dd☐☐ - Oct- 2015

E3. Embarkasi: 1O Banda Aceh; 2O Medan; 3O Jakarta/Bekasi; 4O Makassar/Gorontalo; 5O Banjarmasin/Palangkaraya; 6O Surabaya

E4. Nomor Kelompok terbang / Kloter : ☐☐☐ contoh: 023

F. Paparan Saat Ibadah Haji

F1. Perawatan RS selama di Arab Saudi: 0O Tidak (lanjutkan ke no F2) 1O Ya

F1a. Bila jawaban F1 (ya), lama masa rawat: ☐☐☐ hari 99O Tidak diketahui

F1b. Bila jawaban F1 (ya), atas indikasi: .... 99O Tidak diketahui

F2. Sering mengalami gejala penyakit berikut selama berada di Arab Saudi:

	**F2a.** Gejala penyakit			**F2b.** Jika jawaban F2a (ya), seberapa sering terjadi selama berada di Arab Saudi?		
**1.**	Batuk berdahak	0O Tidak	1O Ya	1O <1 kali	2O 1 kali	3O >1 kali
**2.**	Batuk kering	0O Tidak	1O Ya	1O <1 kali	2O 1 kali	3O >1 kali
**3.**	Demam	0O Tidak	1O Ya	1O <1 kali	2O 1 kali	3O >1 kali
**4.**	Sakit tenggorokan	0O Tidak	1O Ya	1O <1 kali	2O 1 kali	3O >1 kali
**5.**	Pilek	0O Tidak	1O Ya	1O <1 kali	2O 1 kali	3O >1 kali
**6.**	Sakit telinga	0O Tidak	1O Ya	1O <1 kali	2O 1 kali	3O >1 kali

F3. Apakah selama di Arab Saudi, Anda pernah tinggal bersama dengan minimal 1 orang yang menderita infeksi saluran napas/paru? 0O Tidak (lanjutkan ke no F4) 1O Ya

Dikatakan mengalami infeksi saluran napas bila terdapat gejala-gejala batuk, pilek, sakit tenggorok/nyeri menelan, dengan atau tanpa demam
Dikatakan mengalami infeksi paru bila terdapat gejala batuk produktif (berdahak), sesak napas, nyeri dada pleuritik, dengan atau tanpa demam.

F4. Penggunaan antibiotika selama berada di Arab Saudi: 0O Tidak 1O Ya 99O Tidak diketahui

Diisi oleh

Nama personnel : .... Tanda tangan:

Tanggal : dd☐☐ -October -2015

Untuk setiap pertanyaan:
Beri tanda (✓) pada O yang sesuai; atau Isi dengan angka pada ☐; atau Tulis jawaban pada ....
